# Study protocol for a randomised controlled trial to evaluate the use of melanoma surveillance photography to the Improve early detection of MelanomA in ultra-hiGh and high-risk patiEnts (the IMAGE trial)

**DOI:** 10.1186/s13063-023-07203-5

**Published:** 2023-03-29

**Authors:** Mabel K. Yan, Anne E. Cust, H. Peter Soyer, Monika Janda, Katja Loewe, Gabrielle Byars, Paul Fishburn, Paul White, Rashidul Alam Mahumud, Robyn P. M. Saw, Alan Herschtal, Pablo Fernandez-Penas, Pascale Guitera, Rachael L. Morton, John Kelly, Rory Wolfe, Victoria J. Mar

**Affiliations:** 1 School of Public Health and Preventive Medicine, Monash University, 553 St Kilda Road, Melbourne, VIC 3004 Australia; 2 Victorian Melanoma Service, Alfred Hospital, Melbourne, VIC Australia; 3 The Daffodil Centre, The University of Sydney, a Joint Venture With Cancer Council NSW, Sydney, Australia; 4 Melanoma Institute Australia, The University of Sydney, Sydney, NSW Australia; 5 Dermatology Research Centre, The University of Queensland Diamantina Institute, The University of Queensland, Brisbane, QLD Australia; 6 Dermatology Department, Princess Alexandra Hospital, Woolloongabba, QLD Australia; 7 Centre for Health Services Research, The University of Queensland, Brisbane, QLD Australia; 8 Melanoma and Skin Cancer Research Centre, Monash University, Melbourne, VIC Australia; 9 Norwest Skin Cancer Centre, Bella Vista, New South Wales, Australia; 10 NHMRC Clinical Trials Centre, The University of Sydney, Sydney, NSW Australia; 11 Faculty of Medicine and Health, The University of Sydney, Sydney, NSW Australia; 12 Department of Melanoma and Surgical Oncology, Royal Prince Alfred Hospital, Sydney, NSW Australia; 13 Sydney Melanoma Diagnostic Centre, Royal Prince Alfred Hospital, Sydney, NSW Australia

**Keywords:** Total body photography, Melanoma, Surveillance, Early detection, Secondary prevention, Cost–benefit analysis, Randomised controlled trial

## Abstract

**Introduction:**

Melanoma surveillance photography (MSP) is a comprehensive surveillance method that comprises two- or three-dimensional total body photography with tagged digital dermoscopy, performed at prescribed intervals. It has the potential to reduce unnecessary biopsies and enhance early detection of melanoma, but it is not yet standard care for all high-risk patients in Australia. This protocol describes a randomised controlled trial (RCT) designed to evaluate the clinical impact and cost-effectiveness of using MSP for the surveillance of individuals at ultra-high or high risk of melanoma from a health system perspective.

**Methods and design:**

This is a registry-based, unblinded, multi-site, parallel-arm RCT that will be conducted over 3 years. We aim to recruit 580 participants from three Australian states: Victoria, New South Wales and Queensland, via state cancer registries or direct referral from clinicians. Eligible participants within 24 months of a primary cutaneous melanoma diagnosis will be randomised 1:1 to receive either MSP in addition to their routine clinical surveillance (intervention group) or routine clinical surveillance without MSP (control group). Most participants will continue surveillance with their usual care provider, and the frequency of follow-up visits in both groups will depend on the stage of their primary melanoma and risk factors. The primary outcome measure of the study is the number of unnecessary biopsies (i.e. false positives, being cases where a lesion is biopsied due to suspected melanoma on clinical examination, either with or without MSP, but the resulting histopathology finding is negative for melanoma). Secondary outcomes include the evaluation of health economic outcomes, quality of life and patient acceptability. Two sub-studies will explore the benefit of MSP in high-risk patients prior to a melanoma diagnosis and the diagnostic performance of MSP in the teledermatology setting compared to the en face clinical setting.

**Discussion:**

This trial will determine the clinical efficacy, cost-effectiveness and affordability of MSP to facilitate policy decision-making at the national and local levels, across primary and specialist care.

**Trial registration:**

ClinicalTrials.gov NCT04385732. Registered on May 13, 2020.

**Supplementary Information:**

The online version contains supplementary material available at 10.1186/s13063-023-07203-5.

## Background

Skin cancer including melanomas and keratinocyte skin cancers is Australia’s most common cancer. Australia and New Zealand have the highest melanoma incidence rates in the world, and age-standardised rates have doubled in the past 35 years [1]. Despite the less serious nature of keratinocyte cancers, they still cause about 600 deaths and over 100,000 hospitalisations each year [2]. The annual cost of melanoma treatment in Australia in 2017 was estimated to be between AU $1681 and AU $115,109 per patient depending on the stage at diagnosis, with earlier stage associated with improved survival, lower morbidity and lower costs [3]. Thus, secondary prevention of melanoma is important to ensure malignant skin lesions are detected and treated at an early stage when prognosis is better [4], and treatment-related morbidity and healthcare costs are minimised. Improvements in diagnostic performance have the potential to make large savings to the health system and reduce morbidity to patients.

Melanoma surveillance photography (MSP) is a comprehensive surveillance method that combines two-dimensional (2D) or three-dimensional (3D) total body photography (TBP) [5] technology with digital dermoscopy to monitor lesions at set intervals. Baseline images are used as a reference point for skin surveillance. Dermoscopy is associated with a 15-fold improvement in the odds of correctly diagnosing melanocytic lesions as melanoma compared to naked eye assessment [6]. The contribution of 2D or 3D imaging to improved diagnosis is as yet less well studied, but case studies indicate additive value [7].

While there is currently no randomised controlled trial (RCT) evidence that supports the use of MSP for skin surveillance, there is some evidence that specialised melanoma surveillance (incorporating TBP and digital dermoscopy) is a cost-effective strategy for the management of individuals at ultra-high risk of melanoma [8]. In a cohort study conducted over a 10-year period, specialised surveillance through a high-risk clinic was both less expensive and more effective than standard care. The mean saving was AU $6828 (95% CI, 5564 to 8092) per patient, and the mean quality-adjusted life-year gain was 0.31 (95% CI, 0.27 to 0.35) [8]. The main drivers of cost-effectiveness were the detection of melanoma at an earlier stage, resulting in less extensive treatment and better quality of life, and fewer excisions for suspicious lesions with specialised surveillance in the hospital outpatient setting (annual mean rate 0.81; 95% CI, 0.72 to 0.91) compared with standard care in the community (2.55; 95% CI, 2.34 to 2.76). A subsequent budget impact analysis indicated that the Australian healthcare system would save AU $22.6 million over 5 years by using specialised melanoma surveillance incorporating TBP, compared with standard care, for people at very high risk of melanoma [9]. While the evidence has been deemed sufficient to update Australian melanoma clinical practice guidelines [10], MSP is not listed on the Medicare Benefits Schedule because the Medical Services Advisory Committee advised the Minister for Health that the available evidence in relation to comparative safety, clinical effectiveness and cost-effectiveness was not sufficient to support the public funding of MSP [11]. This study will address the critical gaps in the evidence and support the Medical Services Advisory Committee to make an informed recommendation about Medicare Benefits Schedule listing of MSP based on high-quality evidence. Specifically, the study will estimate the extent of health benefit that would be gained from introducing MSP for patients at ultra-high and high risk of melanoma in a variety of settings.

## Objectives

The following is the primary objective:

- Evaluate whether clinical surveillance with MSP, compared to without MSP, reduces the number of unnecessary biopsies (i.e. false positives: where an excision or biopsy of a lesion being performed to diagnose melanoma is identified on histopathology as benign) in ultra-high and high-risk individuals whose risk is contributed to by high naevus counts

The following are the secondary objectives:

1. Assess whether MSP:
  - Reduces “missed diagnoses” (i.e. false negatives) of melanoma
  - Improves the diagnostic performance of all skin cancers (i.e. melanoma combined with keratinocyte cancers)
  - Improves health-related quality of life (HRQoL), patient satisfaction and reduces patient anxiety
2. Determine the cost-effectiveness of the intervention compared with standard care
3. Evaluate the safety and acceptability of MSP
4. Evaluate the diagnostic performance of 2D compared to 3D TBP

The following are the sub-study objectives:

- Evaluate the benefits of MSP prior to a melanoma diagnosis
- Evaluate the diagnostic performance of MSP in a teledermatology setting compared to en face clinical setting.

## Methods and analysis

### Study design and setting

This is an unblinded, multi-centre, parallel-arm RCT. Adults diagnosed with a primary melanoma within 24 months prior to the screening and baseline visit, and who, according to the Melanoma Institute Australia Risk Prediction Tool for Subsequent Primary Melanoma, are at ultra-high or high risk of developing subsequent primary melanoma will be recruited via state cancer registries and direct referral from general practitioners and specialist dermatology settings across metropolitan and regional areas in eastern Australian states [Victoria (VIC), New South Wales (NSW) and Queensland (QLD)]. Identification of participants via the state cancer registries will be limited to postcodes within an approximate 100-km radius of a study site to reduce the screening burden at the registry level and minimise the need for travel. Patients living outside the 100-km radius may be directly referred if they wish to participate. This protocol follows Standard Protocol Items: Recommendations for Interventional Trials (SPIRIT) guidelines (Additional file 1: SPIRIT checklist and Table 2) [12].

### Patient and Public Involvement

This study protocol was developed and endorsed by consumer organisation partners (Melanoma Research Victoria Consumer Reference Group). A consumer representative (P.W.) constitutes part of the investigator team, and their perspectives relating to the conduct of the study, and dissemination of results to wider patient communities are considered.

### Recruitment

**Fig. 1:**
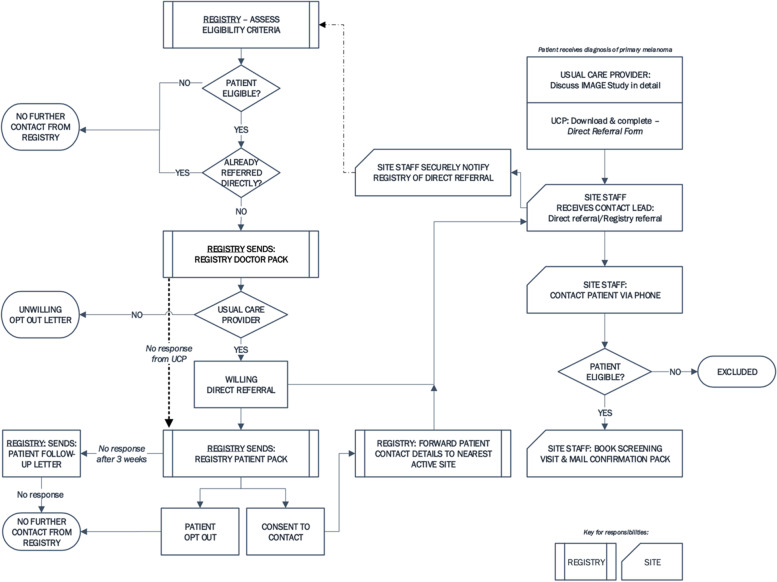
Recruitment schema. Shown is the flow of participants following recruitment directly from the clinician or the state cancer registry

Participants will be recruited via two sources (Fig. 1).

#### Recruitment via direct referral from a treating clinician

Clinicians will be notified of the trial via communications through existing professional networks of investigators and the Melanoma and Skin Cancer Research Centre. Usual care providers may refer a potentially eligible participant directly to the study team. The study team will notify the cancer registry of direct referrals to avoid the patient being re-approached by the registry.

#### Recruitment via state cancer registries

The cancer registry will identify potentially eligible cases, and the clinician who made the diagnosis will be notified. The clinician can then directly refer the patient to the trial or advise the cancer registry if they are deemed ineligible. If no response is received within 3 weeks, the registry will directly notify the patient of the trial. If a patient does not respond to the registry within 1 month, a follow-up letter will be sent. If no response is received, no further attempt at contact will be made.

### Trial eligibility criteria

**Fig. 2:**
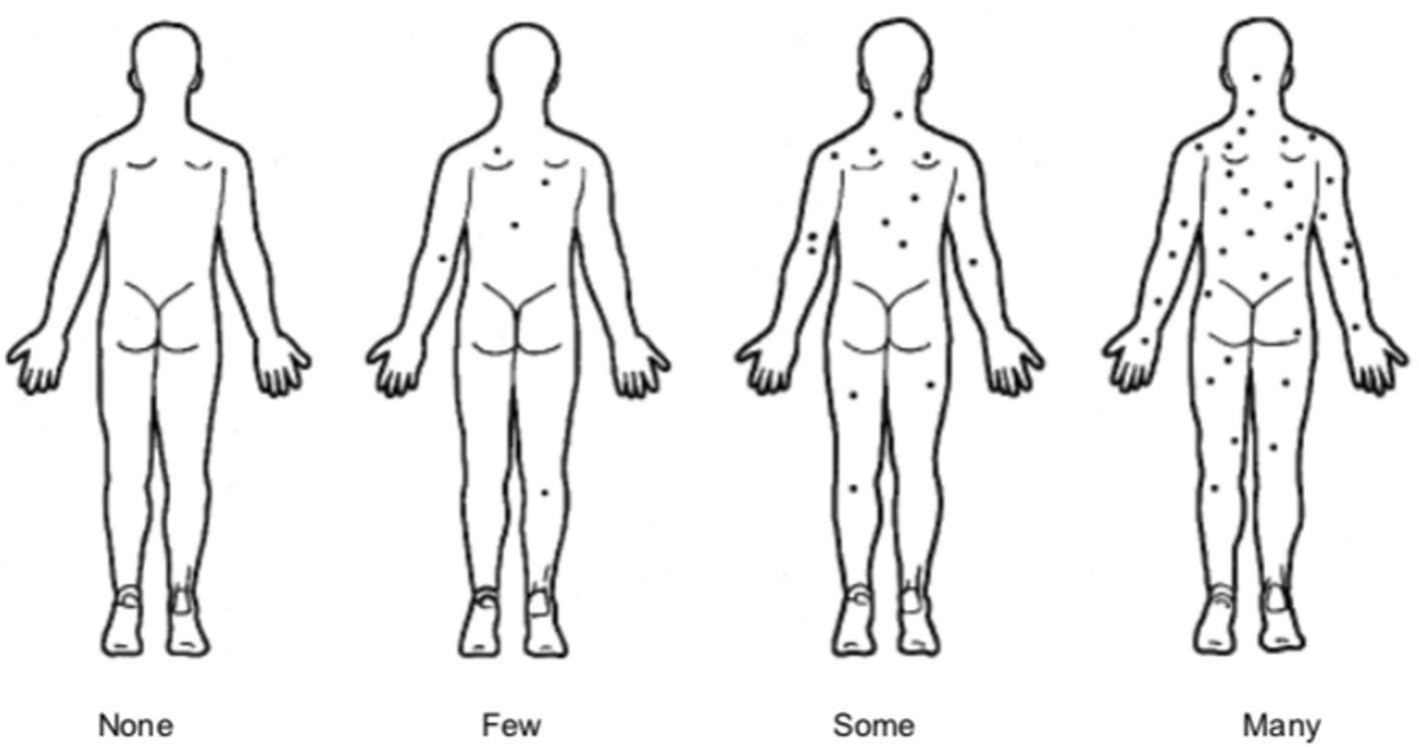
Naevus count estimation pictogram [14]. Shown is the naevus count estimation pictogram used to estimate the naevus count

Individuals may be included in the study if they meet all the following criteria:

1. Aged 18 years or older
2. Within 24 months of the date of a melanoma diagnosis when attending the screening and baseline visit
  - Date of diagnosis refers to the date on the pathology report that provides histological confirmation.
3. Able to provide informed consent, complete questionnaires and attend study site for MSP
4. Appropriate for TBP referral (see the “Section A” section)
5. Ultra-high or high risk of subsequent primary melanoma according to the risk prediction tool (see the “Section B” section) [13, 14]
6. Multiple naevi, as “some” or “many” naevi on the pictogram (Fig. 2)
7. Not previously under active surveillance with TBP for melanoma surveillance (see the “Section C” section)
8. Living in Australia and not planning to move overseas within the next 3 years

#### Section A: Appropriate for TBP referral

Participants are required to be physically able to receive TBP (i.e. able to stand upright unaided). Cancer registries will only contact patients ≤ 75 years of age.

#### Section B: 10-year risk category thresholds

The Melanoma Institute Australia Risk Prediction Tool for Subsequent Primary Melanoma [13] output percentage is converted to a risk category as per below for the purposes of this study:

-  ≥ 20%—ultra-high risk
-  > 5–19%—high risk
-  ≤ 5%—moderate or low risk

#### Section C: Sub-study 1 eligibility

**Fig. 3:**
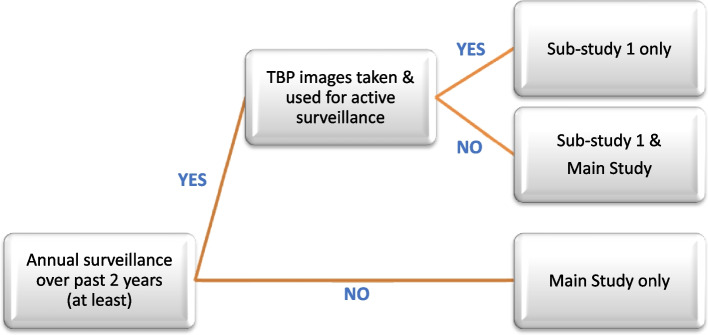
Patient eligibility based on prior surveillance. Shown is a flow chart indicating patient eligibility for the main study and/or sub-study 1

- If a participant meets all eligibility criteria except criteria 7, they are eligible for sub-study 1, and not eligible for the main study. Active surveillance with TBP refers to the acquisition of TBP and its use for melanoma surveillance (see Fig. 3)
- Patients need to have had at least annual skin surveillance over the past 2 years to be eligible for sub-study 1

### Exclusion criteria

Participants will be excluded from the main study for any of the following reasons:

1. Previously under active surveillance with TBP for at least 2 years
2. Stage 4 metastatic melanoma
3. Ocular or mucosal melanoma
4. Participation in another clinical trial or study involving MSP

Note: A past history of melanoma is not an exclusion criterion. Adjuvant radiotherapy and systemic therapies (e.g. cytotoxic, immunotherapy) may be received by participants during the trial.

### Randomisation

The site staff delegated by the principal investigator will undertake the consent processes. Eligibility screening and randomisation will then be undertaken on the Melanoma and Skin Cancer Research Centre web-based randomisation portal. Potential participants will be registered by the delegated site staff on the Melanoma and Skin Cancer Trials website, indicating the study site and date of consent. Participants’ skin will be assessed for the number of naevi (some or many vs few or none) and other risk factors which are entered into the online risk calculator to determine whether the patient’s risk level satisfies the eligibility criterion. If the patient is eligible, the web-based algorithm is applied which assigns the participant randomly to either the intervention or control group. Randomisation is in a 1:1 ratio, stratified by prognostic (ultra-high/high risk of subsequent primary melanoma, sex) and geographical factors (state as VIC/NSW/QLD, residential location as urban/regional to be defined by Australian Bureau of Statistics Remoteness Framework). Within the strata, the randomisation schedule will apply randomly sized permuted blocks of participants. Randomised participants will be notified immediately of treatment arm allocation by site staff.

### Intervention arm

The intervention arm will receive MSP in addition to clinical surveillance with their usual care provider. Baseline MSP will be taken at an approved study site. MSP includes TBP and tagged dermoscopy. Dermoscopic images will be taken of any lesions the participant is concerned about, any lesion the melanographer/nurse practitioner is concerned about and up to 20 naevi that are > 4 mm in diameter. Participants imaged with 3D TBP will have 3D TBP repeated at 12 and 24 months (see substudy 2). Given that 2D TBP can be time- and resource-intensive, repeat annual imaging is not a requirement for participants imaged with 2D TBP. However, follow-up dermoscopic imaging will be taken at 12 and 24 months for all participants in the intervention arm. Dermoscopic imaging of individual lesions may be performed by the treating doctor at more regular intervals if clinically necessary. The study site will maintain a copy of all images, and participants will be provided with their MSP imaging on a password-protected Universal Serial Bus (USB) flash drive if their regular surveillance will be conducted elsewhere. Data on the number of clinic visits, biopsies, procedures and any short-term surveillance photography used will be captured via Medicare Benefits Schedule /Pharmaceutical Benefits Scheme linkage, pathology reports, the participant diary and trial questionnaires.

### Control arm

The control arm will receive clinical surveillance with their usual care practitioner as the standard of care. Data on the number of clinic visits, biopsies, procedures and any short-term surveillance photography used will be captured via Medicare Benefits Schedule/Pharmaceutical Benefits Scheme linkage, pathology reports, participant diary and trial questionnaires. They will be offered a single session of MSP at the end of the trial period and images on a password-protected USB flash drive.

### Primary outcome

The primary outcome is the diagnostic performance of surveillance with MSP compared to standard care without MSP, as measured by the number of unnecessary biopsies (Table 1). The number of biopsies is one of the main drivers of the cost of melanoma surveillance [8].

**Table 1:** Study outcomes

Outcomes	Measure or comparison
**Primary outcome**	
Diagnostic performance for melanoma	Number of unnecessary excisions or biopsies (i.e. false positives). An unnecessary biopsy is one performed due to clinical suspicion of melanoma, where the histopathology is benign
**Secondary outcomes**	
Additional diagnostic performance outcomes for melanoma	NNB to detect one melanoma, benign:malignant ratio Number of false negatives (determined by follow-up) and the morbidity and complications from procedures
Diagnostic performance outcomes for melanoma and other skin cancers combined	Benign to malignant ratio and the NNB
Quality of life	Patient quality of life outcomes—AQOL-8D
Patient anxiety and acceptability	EORTC QLQ-C30, FCR4 and a purpose-designed patient acceptability scale
Health economic outcomes	
- Health system resource utilisation and out-of-pocket costs - Health-related QOL using a preference-based multi-attribute utility instrument - Cost-effectiveness of the intervention compared with control (usual care) - Budget impact analysis	Number of clinic visits, personnel time, procedures, diagnostic tests, hospitalisations, overhead costs (e.g. IT, data transfer systems), capital equipment, consumables and patient out-of-pocket payments documented from Medicare claims AQOL-8D utilities for calculation of QALYs Incremental cost-effectiveness ratios or incremental net benefit for both cost per unnecessary excision or biopsy avoided and cost per QALY gained of MSP compared with usual care Budget impact of the intervention to Australian Medicare

*NNB* number needed to biopsy, *AQOL-8D* Assessment of Quality of Life-8D, *EORTC QLQ-C30* European Organisation for Research and Treatment of Cancer Quality of Life Questionnaire, *FCR4* fear of cancer recurrence–short form, *QALYs* quality-adjusted life years, *MSP* melanoma surveillance photography, *IT* information technology

A skin biopsy or excision may be undertaken if, on clinical examination, a lesion is suspicious for melanoma, or another form of skin cancer, or to diagnose a suspected condition unrelated to skin cancer. Histopathology provides the ground truth diagnosis for biopsied lesions. The primary objective considers a biopsy that reveals malignant histopathology is “necessary”. The full relationship between clinical examination finding, histopathology finding and a designation of false positive, true positive, false negative or true negative can be found in the Statistical Analysis Plan (SAP See supplementary file).

### Secondary outcomes

Secondary outcomes will assess additional diagnostic performance outcomes for all skin cancers (melanoma and keratinocyte cancers), as well as HRQoL, health economic outcomes and patient acceptability of MSP using validated questionnaires at baseline and 12 and 24 months.

### Participant timeline and follow-up procedures

Table 2 and Fig. 3 provide an overview of the participant timeline. All participants will attend a study site for their screening and baseline visit, at which point informed consent, randomisation, clinical assessments and baseline MSP for the intervention arm will occur. If site attendance is restricted (e.g. pandemic restrictions), then this may be performed via video consultation and followed with an on-site visit for MSP when possible (ideally within 1 month). Thereafter, participants will be followed for a 2-year period and have routine regular surveillance visits with their usual care provider. The frequency of these surveillance visits will depend on the stage of the melanoma (as per Cancer Council Australia Melanoma Guidelines: 12-monthly for stages 0–1, 3–6 monthly for stage 2) and risk of subsequent primary melanoma (Cancer Council Australia Melanoma Guidelines specify 6 monthly review for ultra-high risk patients, regardless of melanoma stage, and 6–12 monthly review for high-risk patients). A participant diary will be provided to document skin cancer-related health care visits. At 12 and 24 months, self-reported questionnaires as outlined in Table 2 and assessments administered via a follow-up phone call from study staff will be performed.

**Table 2:**
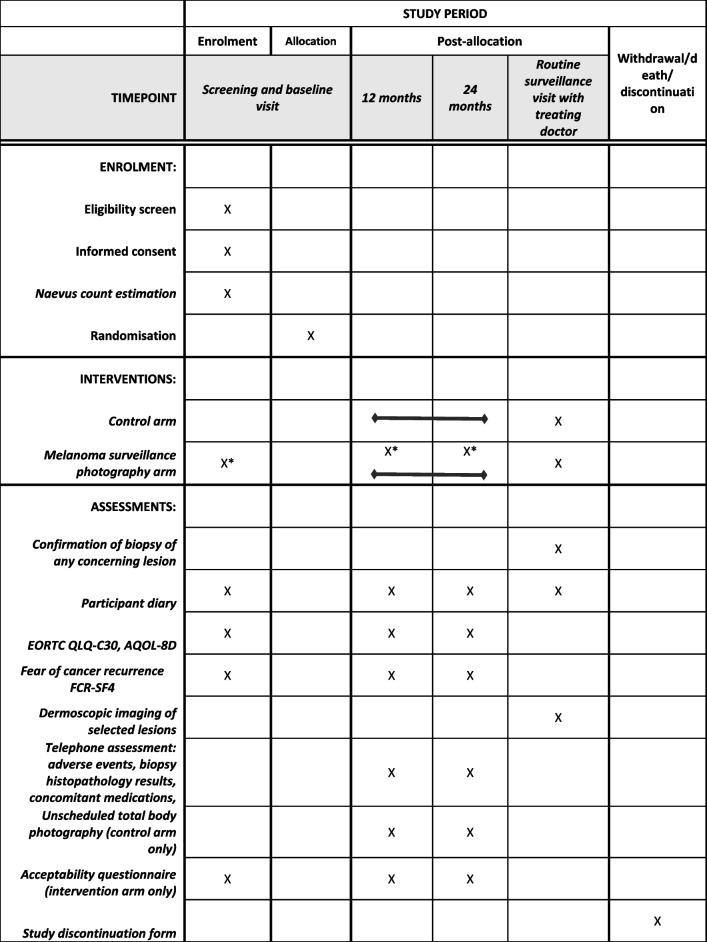
SPIRIT table

^a^Both 2D and 3D total body photography will be performed at baseline. Only 3D total body photography is repeated at 12 and 24 months. All participants will continue routine surveillance with the treating doctor during the trial period

For the intervention arm, annual surveillance visits will include a full skin examination for assessment of the entire skin surface using baseline imaging for comparison. If the participant’s usual care provider is not familiar with the use of MSP for clinical surveillance, they will receive a link to a training video. If no surveillance plan is in place, or if it is the preference of the usual care provider and/or participant, they may be followed up at a study site.

### Withdrawal and loss to follow-up

**Fig. 4:**
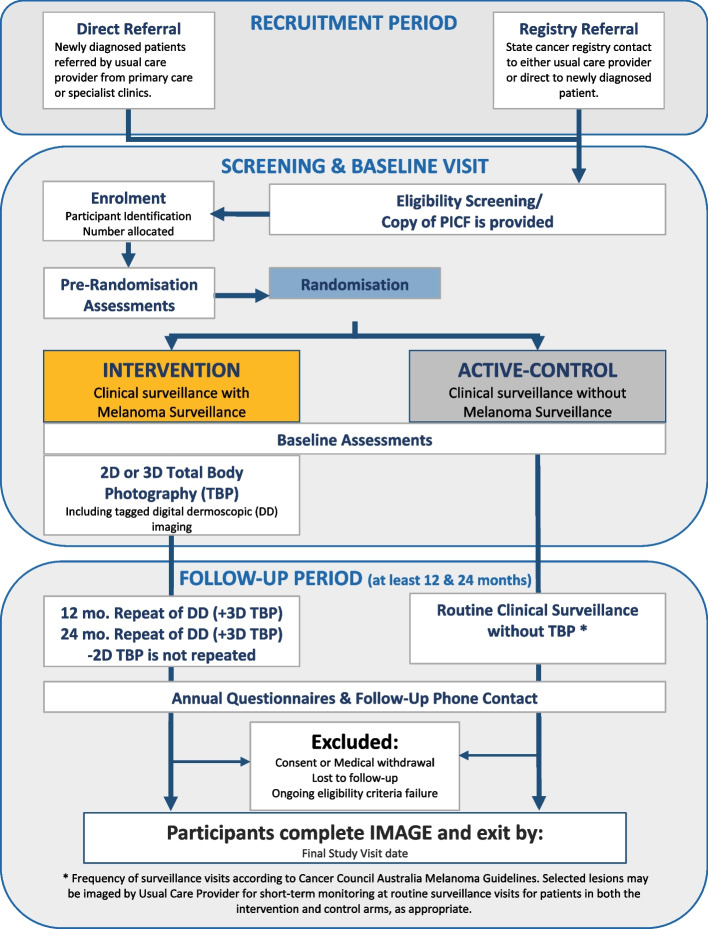
Flow of participants. Shown is a flow chart demonstrating the flow of participants through the trial from recruitment to the final study visit

Participants are able to withdraw at any time without providing a reason (Fig. 4). If a participant decides to stop their follow-up visits but is willing to be contacted via phone, their health status will be ascertained on an annual basis by phone with their usual care provider or site staff. All correspondence regarding the trial will be discontinued for participants who no longer wish to be contacted. A study discontinuation form is to be completed by the site staff and submitted to the Melanoma and Skin Cancer Research Centre.

### Data collection and management

Melanoma and Skin Cancer Research Centre staff will maintain the trial data. Data collection forms are stored electronically on REDCap (hosted by Monash University). Electronic versions of printable CRFs are stored in the TMF, managed by Melanoma and Skin Cancer Research Centre.

Prospectively collected trial data, and data collected through participant diaries at trial completion, is entered directly into the IMAGE Main Study REDCap database. Separate REDCap databases will house participant data relevant to trial sub-studies. Three-dimensional TBP with tagged dermoscopic images will be generated by VECTRA® WB360 medical imaging management systems. Images and clinical information are processed on site within proprietary, Digital Imaging and Communications in Medicine-compliant DermaGraphix software developed by Canfield Scientific Inc. (Parsippany, NJ, USA). Two-dimensional TBP and dermoscopic images will be generated by standard 2D imaging equipment available at each site.

Images and clinical data will be securely stored at each site’s Picture Archiving and Communication System, Electronic Medical Record or Vendor Neutral Archive hosted within the Information Technology infrastructure of the relevant health service. Security and data privacy specifications are site-specific and meet relevant local, state and federal requirements for health information management. Participant data will be collected, stored, managed and accessed by approved research personnel on Monash University’s purpose-built secure eResearch Data Storage platforms.

### Data monitoring

Data quality is maintained by the Melanoma and Skin Cancer Research Centre via central and on-site monitoring. Each site involved in the trial received protocol-specific training and melanography training prior to commencing recruitment. Additionally, a central data review is performed on an ongoing basis to query any illogical, missing or contradicting with sites and followed up until resolution as well as annual on-site monitoring visits to ensure high-quality data and adherence to the protocol and GCP.

The Trial Management Committee (TMC) determined that an independent Data Safety and Monitoring Board was not required given the low-risk MSP posed to participants, based on observational data that photography as an aid during clinical examination is superior to standard care. However, regular TMC and Operations meetings are conducted to address any issues pertaining to data quality and participant safety. The study team will discuss any changes to the study protocol, and if amendments are required, they will be approved by the HREC before implementation.

### Adverse events

Adverse events are defined as any untoward medical occurrence in a trial participant and will be collected at follow-up visits (12 and 24 months), until trial completion, withdrawal, lost to follow-up or death. Data around the occurrence of skin cancer-related adverse events (wound infection or dehiscence) from biopsies and procedures will be captured.

### Site audits

This trial is subject to audit by each of the stakeholder groups involved and relevant regulatory authorities. Where possible, sites will be informed in writing by Melanoma and Skin Cancer Research Centre or the relevant external stakeholder, and the scope of the audit will be outlined.

## Statistical methods

### Sample size

In standard care without MSP, high-risk patients have on average 2.0 biopsies per year [8]. With MSP, we expect this to reduce by 25% to a mean of 1.5 per year. The distribution of the number of excisions per high risk patient does not follow a normal distribution, nor is it expected to follow a Poisson distribution, and thus will be assumed to follow a negative binomial distribution. We assume for the purpose of sample size estimation, that the sample size will be large enough for the mean to be normally distributed in accordance with the central limit theorem. Estimation of within group variances are based on the negative binomial distribution with a mean of 2.0 and a zero count of 35% in the control arm, compared with a mean of 1.5 and a zero count of 41.5% in the MSP arm.

Given that access to 3D TBP is expected to improve over the next 3 years, we anticipate a cross-over of up to 20% of patients from the control arm to MSP. Thus, under the intention-to-treat (ITT) principle, in which patients are analysed as part of the arm they were originally assigned, the effective mean biopsy rate in the control arm considering cross-over reduces from 2.0 per year to 2.0 × 0.8 + 1.5 × 0.2 = 1.9 per year.

In an ITT analysis, based on an assumption of a benign to malignant ratio (i.e. false-positive to true-positive ratio) of 3.5 to 1 in the control arm and identification of the same number of true positives in the control and MSP arms, to have 80% power to detect a difference between standard care and MSP in mean excisions per high-risk patient of 1.9 per year (standard care) v 1.5 per year (MSP), with excisions counted over two years, we require 245 patients per arm. Although approximately 15% loss to follow-up is anticipated, it will be possible to access biopsy counts for patients lost to follow-up from Medicare Benefits Schedule linkage data, so the estimated required sample size is still 245 × 2 = 490 participants. In the absence of an interim analysis, the initial planned sample size will incorporate a margin of error and will be set to 580 participants in total. This sample size may be reviewed pending the outcome of a pre-planned interim analysis.

### Interim analyses

A formal interim analysis will be performed if accrual by 30 October 2022 is below 400. The mean and variance of the number of excisions per patient in the standard care arm will be estimated from the standard care participants accrued up until this point and used to revise the final sample size.

### Statistical analyses of primary endpoint

The number of lesions identified as suspicious and biopsied over 2 years starting from the date of randomisation will be counted for each patient. Histopathology will confirm each biopsied lesion as either a true positive or false positive. An independent groups *t*-test will be used to compare the mean number of false-positive findings per participant between the standard care and MSP arms. The mean and 95% CI of this difference and the corresponding *p*-value for the null hypothesis of no difference between arms will be provided. The number needed to biopsy (NNB), calculated by dividing the total number of biopsies performed by the number of biopsies which turned out to be true positives, will be compared descriptively between arms (the NNB is the inverse of the positive predictive value, the PPV).

### Analytical strategies for secondary endpoints

#### Additional diagnostic performance outcomes for melanoma

The benign to malignant ratio (B:M = NNB − 1) and false-negative rate (missed diagnoses) will be compared descriptively between the standard care and MSP arms. Accurate estimation of false negative rates (i.e. a missed melanoma) is of interest in order to reassure us that any measured difference in the NNE between the MSP and non-MSP arms is not offset by an increase in missed diagnoses. All cases of invasive melanoma will be referred to a blinded adjudication committee, who will determine whether, based on tumour thickness, frequency of clinical visits and lesion history, the melanoma would likely have been observable on the prior clinical assessment, but was missed (i.e. a false negative). An in situ melanoma or keratinocyte cancer diagnosed at a subsequent visit will not be considered sufficient grounds for an assessment of a missed diagnosis. All lesions that remain unbiopsied after a period of at least 3 months will be assumed true negative. Given it is impractical and imprecise to record total naevus counts for each individual, true negatives will not be formally reported at a lesion level. A more detailed description of how a negative clinical examination finding can be determined to be a false negative can be found in the SAP (See supplementary file).

Diagnostic performance endpoints will consider all biopsies performed during the first 2 years after randomisation, consistent with the primary objective. Pre-specified sub-group comparisons of diagnostic performance will be undertaken by: risk level (ultra-high risk/high risk), imaging modality (2D/3D imaging), location (regional/urban) and care setting (primary care/specialist) by repeating the analysis for the primary objective in these sub-groups.

#### Diagnostic performance for all skin cancers

Calculation of the number of biopsies performed to diagnose all skin cancers (melanoma and other skin cancers combined) per patient, the benign to malignant ratio and NNB will be compared descriptively between the standard care and MSP arms. Comparisons of diagnostic performance will be undertaken in the sub-groups specified above. False negatives and true negatives will not be adjudicated for keratinocyte cancers.

#### Quality of life

The EORTC QLQ C30 will be scored centrally according to the EORTC QOL scoring manual [15]. For each scale of interest, the mean and standard deviation of the score across patients at each assessment time point (screening and baseline visit, 12 months after the screening and baseline visit and 24 months after the screening and baseline visit) will be presented and compared to Australian normative reference values published in 2019 [16]. The AQOL-8D has eight subscales and can be scored as a HRQoL instrument and as a utility instrument for health economic analyses. It will be assessed at the same time points as the QLQ C30. Patient-reported quality of life outcomes will be measured as change in HRQoL parameters during the course of treatment. Quality of life will be compared between treatment groups using a mixed effects linear regression model to compare change from baseline between the two arms. Assessment time point and arm will be fixed effects and participant will be a random effect. Baseline quality of life will be controlled for. The proportion of patients who experience a greater than 5% or 10% reduction in quality of life or fear of recurrence from baseline will also be summarised for each arm at each time point and compared between arms at each time point using a mixed effects logistic regression model. The fixed and random effects to be included will be the same as for the mixed effects linear regression above.

The pattern of missing healthcare use and utility-based quality of life data required for the economic evaluation will be assessed and tabulated. The appropriate method for handling missing data will depend on the proportion of missing data and likely mechanism of missingness (missing not at random (MNAR), missing at random (MAR)) [17]. Multiple imputation methods may be used if healthcare use data or AQOL-8D data are MAR, using a method considered most appropriate at time of analysis.

#### Potential sources of bias

We anticipate that participants in the control arm, who are not coming back to sites for imaging and have follow-up “standard care” visits scheduled in the community may have incomplete or missing 12 and/or 24 month questionnaire data. The use of a mixed effects linear regression will allow inclusion in the model of data from patients who have either dropped out or have intermittent missing data. So as long as the missingness pattern is either missing at random or missing completely at random (MAR or MCAR), inferences will be unbiased by the missingness and require no separate missing data imputation method. A compound symmetry correlation structure will be used to account for repeated measures from the same individual. The potential for missing biopsy data will be minimised by linkage with Medicare Benefits Scheme item numbers for skin biopsies.

To reduce bias, all analyses of the final data set will be performed blinded to the study arm. To minimise bias when assessing diagnoses and possible missed diagnoses, adjudicators will also be kept blinded to the study arm. Longer-term assessment of melanoma incidence beyond the study period will be performed via data linkage with cancer registries and can also be used to adjudicate possible missed diagnoses.

A detailed SAP is described in the supplementary file.

#### Economic evaluation

To assess the economic value of within-trial outcomes for MSP from an Australian health system perspective, the economic analysis will be performed in terms of “unnecessary excision or biopsy avoided” using individual patient-level data from this trial. The mean number of excisions/biopsies avoided will be assessed for both groups at 24 months. Healthcare utilisation will be assessed through study records, participant diaries and through linked Medicare Benefits Schedule and Pharmaceutical Benefits Scheme claims and admitted patient data through the Centre for Health Record Linkage (CHeReL). The second economic outcome is quality-adjusted survival, measured in quality-adjusted life-years (QALYs) gained, which will be applied in a longer-term economic model. HRQoL will be calculated from the AQOL-8D utilities collected within the trial, using baseline and 12- and 24-month measures. Using a health state Markov model, utilities will be combined with survival data to estimate QALYs. The analytical approaches will take the form of cost-effectiveness and cost-utility analyses. Based on trial evidence, incremental cost-effectiveness (and cost-utility) ratios will be calculated by taking a ratio of the difference in the mean costs and mean effects. An incremental net benefit may also be calculated. Uncertainty will be assessed through a scenario, one-way and probabilistic sensitivity analyses. A budget impact analysis similar to our prior study [9] will estimate the affordability of MSP to the Australian healthcare system and Medicare at scale. Technical details about the economic evaluation will be documented in the Health Economics Analysis Plan (HEAP).

## Planned sub-studies

Two planned sub-studies will assess selected outcomes.

### Sub-study 1—Pre-trial excision rates

To assess the benefit of MSP in high-risk patients before a melanoma diagnosis, this study will compare Breslow thickness of the primary melanoma at trial entry and biopsy rates prior to diagnosis between participants who were under at least annual surveillance:

- With TBP for at least a 2-year period prior to their melanoma diagnosis
- *Without* TBP prior to their melanoma diagnosis, for at least a 2-year period prior to their melanoma diagnosis

This will involve linkage with Medicare Benefits Schedule data and will include the collection of pathology reports over a time frame of up to 5 years prior to study enrolment. Data will be collected on the frequency of skin checks, doctor performing skin examinations and tools used by that doctor. This will also involve a telephone interview, Medicare Benefits Schedule linkage and verification via telephone contact with previous practices. Patients who are not trial eligible will be invited to participate in this sub-study with a separate consent form. The Breslow thickness of the primary melanoma and excision rates will be compared descriptively between the groups by providing the mean, SD, median and inter-quartile range for each group. Excision rates will be weighted in proportion to the duration of time over which the excision rate was measured.

### Sub-study 2—Telediagnosis validation

Approximately 100 paired sets of 3D TBP and tagged dermoscopic images (baseline and follow-up) taken from participants in the intervention arm will be sent to 10 study doctors for teledermatology assessment. Images will be selected randomly for this validation study. Approximately 30% of these will include an image of a melanoma confirmed by histopathology (gold standard) during the trial. This sub-study will assess the diagnostic performance (true positive, false-positive and false-negative rates) in the teledermatology compared to the en face setting to validate its use in routine care. Telediagnosis has the potential to improve access, particularly for rural patients and reduce travel and costs associated with surveillance visits. Images may also be assessed by artificial intelligence algorithms to compare the sensitivity and specificity of artificial intelligence diagnosis compared to clinicians’ diagnosis.

## Discussion

The robust RCT design including a sufficiently large sample size of this study will provide high-quality evidence to allow critical evaluation of the effectiveness of TBP for melanoma surveillance amongst those at ultra-high or high risk of melanoma. By recruiting eligible cases through direct referral and state cancer registries, this registry-based RCT combines the advantages of randomisation with the benefits of large-scale population-based registry data and aims to achieve population sampling of participants under the surveillance of different health professionals, as well as from regional and metropolitan areas. Most participants will continue surveillance with their usual care providers and multiple data outcomes will be collected via data linkage to national databases, foregoing the need for intensive trial follow-up visits and reflecting a more pragmatic design more closely aligned to “real-world” settings, thus enhancing the generalisability of study results. However, the study may not be generalisable to patients at lower risk of melanoma or with low naevus counts, since they are not eligible for this study.

## Impact

This trial will determine the clinical efficacy, cost-effectiveness and affordability of MSP to facilitate policy decision-making at Commonwealth (i.e. for the Medical Services Advisory Committee) and local levels, across primary and specialist care.

## Trial status

The IMAGE trial commenced recruitment in March 2021. Protocol version 4.0, 13 December 2021.

## Supplementary Information

**Additional file 1.** SPIRIT Checklist*.*

**Additional file 2.** MSP to improve early detection of high-risk melanoma.

**Additional file 3.** Supplementary Table.

## Data Availability

The datasets analysed during the current study and statistical code are available from the corresponding author on reasonable request, as is the full protocol.

## Abbreviations

MSP: Melanoma surveillance photography
RCT: Randomised controlled trial
2D: Two-dimensional
3D: Three-dimensional
TBP: Total body photography
HRQoL: Health-related quality of life
VIC: Victoria
NSW: New South Wales
QLD: Queensland
USB: Universal Serial Bus
NNB: Number needed to biopsy
AQOL-8D: Assessment of Quality of Life-8D
EORTC QLQ-C30: European Organisation for Research and Treatment of Cancer Quality of Life Questionnaire
FCR4: Fear of cancer recurrence – short form
QALYs: Quality-adjusted life years
TMC: Trial management committee
ITT: Intention-to-treat
CI: Confidence interval
