# Supplementary material for: Study protocol for a randomised controlled trial to evaluate the use of melanoma surveillance photography to the Improve early detection of MelanomA in ultra-hiGh and high-risk patiEnts (the IMAGE trial)
Source: Trials. 2023 Mar 29;24:236. doi: 10.1186/s13063-023-07203-5 (PMC10061902; doi:10.1186/s13063-023-07203-5)
Supplement: Supplementary file 2 — Additional file 2. MSP to improve early detection of high-risk melanoma. [file 13063_2023_7203_MOESM2_ESM.docx]

**MSP to improve early detection of high-risk melanoma**

Melanoma surveillance photography (MSP) to Improve early detection of MelanomA in ultra-hiGh and high-risk patiEnts

**Statistical Analysis Plan**

**IMAGE (02.19)**

Version 1.1 dated 22^nd^ February 2023

Authors: Alan Herschtal, Rory Wolfe

1. BACKGROUND

Skin cancer is Australia’s most common and most expensive cancer. Each year, in Australia, there are about 14,000 new cases of invasive cutaneous melanoma (4^th^ most common cancer) and over 1,800 melanoma deaths. Australia and New Zealand have the highest melanoma rates in the world and age-standardised rates have almost doubled in the past 35 years. Further, there are just as many new cases of melanoma in situ as there are of invasive melanoma. Further, the incidence of the more common keratinocyte cancers is higher than that of all other cancers combined, with two-thirds of Australians diagnosed in their lifetime. Although less serious, keratinocyte cancers still cause about 600 deaths and over 100,000 hospitalisations each year, representing one-quarter of all cancer-related hospitalisations, the most of any cancer. The annual cost of melanoma treatment is estimated to be as high as $115,109 per patient depending mostly on stage at diagnosis. For these reasons, secondary prevention of skin cancer, particularly melanoma, is important to ensure that any malignant skin lesions are detected and treated at an early stage when prognosis is better, and that treatment-related morbidity and healthcare costs are minimized. The sheer volume of benign lesions that are excised unnecessarily because of imperfect diagnostic tools in the effort to diagnose the malignant skin cancers contributes almost half of total cost. Improvements in diagnostic performance have the potential to make large savings to the health system and to reduce morbidity to patients. Given the magnitude of healthcare spending, the Australian Government needs better evidence to make informed decisions about reimbursement of new investigative services.

Melanoma Surveillance Photography (MSP) may improve health outcomes and reduce health system costs. Research has shown that specialised melanoma surveillance (incorporating total body photography (TBP) and digital dermoscopy (DD) is a cost-effective strategy for the management of individuals at very high risk of melanoma. This study will address the critical gaps in evidence for comparative safety, clinical and cost effectiveness of MSP

1. ENDPOINTS
   1. Primary Endpoint

The primary endpoint is the number of false positive biopsies (unnecessary biopsies) per participant over the course of the 2-year follow-up period.

During their course of treatment, patients undergo a series of clinical examinations which may indicate that a biopsy is required to check for suspected melanoma. A false positive is a biopsy taken after clinical examination which identified a lesion was suspicious for melanoma, but where the histopathology was negative for melanoma.

- 1. Secondary Endpoints

1. **Number Needed to Excise (NNE)**

The NNE is the number of lesions biopsied due to clinical suspicion, for each true positive identified on histopathology. It may be defined as the total number of lesions biopsied (true positives plus false positives) divided by the number of true positives, (TP + FP)/TP, or alternatively, as the inverse of the probability of a positive finding on biopsy given a positive finding (suspected melanoma) on clinical examination. This quantity will be estimated within each arm. The definition of a true positive can be found below. False positives are as defined for the primary endpoint.

1. **Benign to Malignant Ratio**

The benign to malignant ratio will also be reported. It can be derived as a simple transformation of the NNE, namely the benign to malignant ratio = NNE – 1.

1. **True Positive (and False Positive) Findings**

A true positive finding is one that results from a lesion suspicious for melanoma on clinical examination, and for which that suspicion was confirmed histopathologically on biopsy. (False positive biopsies are defined above as part of the description of the primary endpoint.)

There will be multiple clinical visits per patient (following randomisation at baseline study visit), and each patient will have multiple naevi and other skin lesions assessed per visit. The method of determining whether a finding is true positive or false positive differs slightly between the first visit and subsequent visits, as per below.

***On first clinical visit (following randomisation)***

If clinical examination (with or without MSP, depending on the treatment arm) identifies a lesion suspicious for melanoma, that lesion will be biopsied. For a patient’s first clinic visit, a histopathology finding which confirms the positive clinical examination finding will be recorded as a true positive. On the other hand, a histopathology finding which fails to confirm the positive clinical examination finding will be recorded as a false positive.

If clinical examination (with or without MSP) does not identify any lesions suspicious for melanoma, then no biopsy is undertaken. Based on the clinical examination alone, it is not possible to ascertain whether this negative finding is a true negative or a false negative, but in the absence of any other evidence, it will be assumed to be a true negative. However, figure 3 below describes how a ‘true negative’ finding may be overturned into a false negative at a subsequent visit. Figure 1 shows the sequences of events leading to a true positive (or to a false positive) finding on a first visit. (For all flowcharts, the current visit is shown in dark grey, with previous or subsequent visits shown in light grey.)

P

P

= clinical examination

= histopathology assessment

**Figure 1**: True positive and false positive on first clinical visit (P= positive finding, N = negative finding)

***On subsequent clinical visits***

On subsequent clinic visits, essentially the same process will be followed. The sequences of events for a true positive (first flowchart) and a false positive (second flowchart) on a subsequent visit are shown in figure 2.

P

P

**Figure 2**: True positive and false positive on a subsequent clinical visit (P= positive finding, N = negative finding)

However, on visits subsequent to the first, it is possible that an earlier visit, previously assumed to be a true negative in the absence of any evidence to the contrary, could be re-evaluated as a false negative. This can happen if there is a positive finding on histopathology from the visit that is adjudicated as being ‘missed’ at the preceding visit. The sequence of events corresponding to this scenario is shown in figure 3.

P

N

**Figure 3**: Re-evaluation of a true negative to a false negative due to a later positive histopathology finding (P= positive finding, N = negative finding, - = no biopsy taken)

Because the clinical examination was not suspicious (or was overlooked) for invasive melanoma at the first visit, no biopsy was performed and hence no histopathology finding was available. Thus, the negative finding was provisionally assumed to be a true negative. On the subsequent visit, however, where a suspicious finding on clinical examination was confirmed by a positive finding on histopathology, the question arises as to whether that positive finding should have been observable at the preceding visit, but was missed. Such cases will be referred to a blinded adjudication committee, who will determine, based on tumor thickness, frequency of clinical visits and lesion history, whether the melanoma would likely have been observable on the prior clinical assessment, but was missed. Such ‘missed diagnoses’ are false negatives.

An in-situ melanoma or keratinocyte cancer diagnosed at one of these subsequent visits will not be considered sufficient grounds for an assessment of a missed diagnosis. Provided there is no subsequent biopsy after a follow-up period of at least 3 months, all unbiopsied lesions will be assumed to be true negative. (It is anticipated that missed diagnoses [false negative findings] will be rare.)

1. **True Negative and False Negative Findings**

True negative findings are very difficult to quantify because many patients will have a very large number of benign naevi (possibly many hundreds) which are of no clinical concern. All of these are negatives, but because negative findings on clinical examination are not biopsied, histopathology results establishing whether these are true negatives or false negatives in most cases will not be available. Occasionally, a lesion which is not suspicious on clinical examination will be biopsied. This can happen for one of two reasons. First, it is possible that a biopsy be indicated for a reason other than a suspected malignancy. If such a biopsy shows melanoma then this constitutes a false negative finding. The other way in which a false negative finding can be determined is by re-evaluation of an earlier negative finding, as depicted in the flowchart in figure 3 above.

1. TRIAL DESIGN & RANDOMISATION

The trial employs stratified randomization (see details below). It is a multi-centre superiority study seeking to compare the rate of unnecessary biopsies between patients receiving MSP and those receiving standard of care (clinical surveillance without MSP).

- 1. Randomisation and Stratification

Eligible participants will be randomised in a 1:1 ratio to either the intervention group (MSP) or the control group (usual care). Randomization will be stratified by anticipated prognostic factors (high vs. very-high risk of subsequent primary melanoma and sex) and geographical factors (state as NSW/QLD/VIC, residential location as urban/regional to be defined by Australian Bureau of Statistics Remoteness Framework). Within strata, the randomisation schedule will apply randomly sized permuted blocks of participants. The trial is unblinded, and randomised participants will be notified immediately of their treatment arm allocation by site staff.

1. OBJECTIVES & STATISTICAL CONSIDERATIONS
   1. Basic Descriptive Statistics

Baseline demographic characteristics (age, sex, geographic state and urban/regional status) and disease related characteristics (risk level, Breslow thickness at randomization) will be summarised by study arm and overall, using the methods described for continuous or categorical valued variables below.

Continuous valued characteristics will be summarised by number non-missing, mean, standard deviation, median and inter-quartile range (IQR).

Ordinal and categorical valued characteristics will be summarised by total non-missing and by counts and percentages at each level of the variable being summarised. Percentages will be calculated using the number of non-missing values as the denominator.

No significance tests for a difference between arms in baseline characteristics will be performed.

- 1. Safety

AE’s will be tabulated per treatment arm by type and grade using the grading system CTCAE (v5.0), considering the worst grade only per AE type per patient.

- 1. Objectives

***Primary Objective Definition***

The primary objective of this study is to evaluate, amongst the population of interest, whether melanoma surveillance with MSP, comprising either 2D or 3D TBP tagged with digital dermoscopy (DD), compared to clinical surveillance without MSP, results in a reduced number of unnecessary biopsies. The population of interest is individuals with high or very high risk of melanoma, whose risk is contributed to by high naevus counts.

***Secondary Objectives Definition***

1. **Number Needed to Excise (NNE)**

To estimate the NNE within each arm (mean and SD), and test for a difference in the NNE between arms.

1. **Benign to Malignant Ratio**

To estimate the benign to malignant ratio within each arm (mean and SD). Since the benign to malignant ratio = NNE – 1, then the test for a difference in the benign to malignant ratio between arms is identical to the test for a difference in NNE between arms.

1. **True positives and False Positives**

To provide an estimate of the rate of true positive and false positive findings for melanoma in each arm.

An estimate of the true positive rate is required in order to establish that any observed improvement (reduction) in the unnecessary biopsy (false positive) rate (primary objective), or improvement in the NNE (1^st^ secondary objective) is not due simply to a tighter definition of what is considered suspicious for melanoma under MSP (i.e. less conservative, or less likely to flag a lesion as suspicious). If MSP is less conservative than standard clinical surveillance in aiding identification of a lesion as suspicious, this will result in a reduced false positive rate, and hence possibly a conclusion of a difference between arms in favour of MSP on the primary objective, but at the expense of a lower true positive rate. (See also the sample size calculation section below, which is based on assuming that the true positive rates are the same in both arms.)

1. **False negatives**

To estimate the false negative rate within each arm. The false negative rate provides insight into the sensitivity within each arm, (sensitivity = true positive/(false negative + true positive)).

- 1. Statistical Considerations

***Primary Objective Statistical Method***

An independent groups t-test will be used to compare the mean number of false positive histopathology findings per participant between arms. for each participant, this will be the aggregate of all false positive findings across all visits for the entire 2-year study period. The mean and 95% CI of this difference will be provided and the p-value for the hypothesis test with null hypothesis of equal false positive rates will be provided.

Prior to analysis, presence of heteroscedasticity will be assessed graphically. If substantial heteroscedasticity is suspected, consideration will be given to applying a variance stabilising transformation (i.e. a log transformation) prior to analysis.

Although the outcome measure for this objective (the number of false positive histopathology findings) is a count, for which count modelling methods such as Poisson regression and negative binomial regression would be appropriate, due to the large sample size, we invoke the CLT and analyse using a t-test.

***Secondary Objectives Statistical Methods***

1. **NNE**

Due to the hierarchical nature of the data (lesions nested within visits nested within patients) the following method will be employed to estimate the NNE.

A mixed effects logistic regression model will be constructed. Modelling will be performed at the lesion level, using only those findings which were positive on clinical examination. The histopathological finding (gold standard) will be the dependent variable and treatment arm (surveillance with MSP vs surveillance without MSP) will be the fixed effect (independent variable). Patient ID will be included as a random effect. The model will be used to estimate the probability of a positive histopathology finding given a positive finding on clinical examination for each of the arms, and their 95% CI’s. These figures constitute the positive predictive value (PPV) for each arm. The NNE’s and corresponding 95% CI’s will then be estimated by inverting the PPV’s, since NNE = 1/PPV.

1. **Benign to Malignant Ratio**

The mean and 95% CI of the benign to malignant ratio within each arm will be estimated by simply subtracting 1 from the corresponding NNE estimates above.

1. **True negatives, missed diagnoses, and true positives**

At each visit for each treatment arm, the following will be provided (descriptive statistics only):

1. the count of patients with all lesions true negative;
2. the count of patients with at least one missed diagnosis (at least one false negative)
3. the total number of missed diagnoses (false negatives) across all patients
4. the total number of true positive findings across all patients
   1. Power and Sample Size Calculation

In standard care without MSP, high risk patients have on average 2.5 excisions per year. With MSP, we expect this to reduce by 25% to a mean of 1.875 per year. The distribution of the number of excisions per high risk patient does not follow a normal distribution, nor Poisson, and is unlikely even to be negative binomial. Nevertheless, we assume that the central limit theorem can be relied upon to estimate the difference in means between treatment arms and thus that a two-sample z-test with type 1 error rate of 5% can form the basis of an approximate calculation given the large sample size that will be required. In the original sample size calculation provided in the trial protocol, the standard deviation for this approximate calculation was assumed to come from a negative binomial model, for simplicity and due to a lack of detailed data on the exact distributional form of number of excisions per patient. An interim analysis using updated external data, suggested zero-inflation in the distribution of counts. Thus, an updated sample size calculation used a standard deviation given by an assumption of an underlying zero-inflated Poisson distribution (i.e. a mixture of a Poisson with zeroes with variance=mean in the Poisson component) with 30% zero inflation.

Given access to high quality efficient 3D imaging technology will be significantly improved over the next 3 years with the Australian Cancer Research Foundation’s ACEMID initiative, we anticipate cross-over of 20% of patients from the control group to MSP. Based on the assumptions above, this would be expected to reduce the observed mean excisions in the control group to 2.4375.

In an intention to treat analysis, based on an assumption of a benign to malignant ratio (i.e. false positive to true positive ratio) of 3.5 to 1 in the standard care arm and identification of the same number of true positives in the standard care and MSP arms, to have 80% power to detect in an intention to treat analysis, a difference between standard care and MSP in mean excisions of 2.4375 vs 1.875, we require 289 patients per treatment arm which is in keeping with the original sample size target of 580.

- 1. Missing data and bias

The potentially very large number of benign lesions per patient which are of no clinical concern (true negatives) makes it very difficult to arrive at a precise true negative count. However, the objectives chosen (unnecessary biopsies, NNE, benign to malignant ratio) are not dependent on precise knowledge of the true negative count, so this is not a major limitation.

- 1. Other Statistical Issues

The differential diagnosis for melanoma on clinical examination is frequently a benign melanocytic naevus. The intervention (photography) provides objective evidence of whether a naevus is stable (likely benign) or changing (possibly malignant). The literature often discusses how many benign lesions (comprised mostly of benign naevi) need to be excised to diagnose 1 melanoma. This is often quoted as approximately 3:1 in specialist settings, and up to 30:1 in general practice settings.

Some patients have a very high burden of keratinocyte cancers (BCC, SCC) which require biopsy / excision. These more indolent tumors are typically easier to diagnose clinically and outcomes are not thought to be impacted significantly by Melanoma Surveillance Photography.

We will therefore perform 2 separate analyses, one which considers melanoma in isolation and a second which considers all skin cancers combined, as outlined below.

In designing the mapping from the clinical examination and histopathology findings to a designation of one of TP, FP, FN, or TN we need to ask two questions:

1. What constitutes a positive finding on clinical examination? A) a biopsy taken to confirm a suspected/possible melanoma, or B) a biopsy taken to confirm either melanoma or another skin cancer (BCC/SCC)?
2. What makes a positive finding on clinical examination a true positive on histopathology? A) Just the case where histopathology showed melanoma, or B) any case where histopathology showed either melanoma or other skin cancer?

**Melanoma specific analysis**

In this analysis, we are interested in any cases where the histopathology confirms melanoma (regardless of clinical suspicion) AND cases where a biopsy is taken because melanoma is suspected (but histopathology is benign)

| **Result of clinical examination** | **Histopathology finding** | |
| --- | --- | --- |
|  | **Melanoma** | **Benign** |
| **Melanoma suspected** (biopsy taken)** | TP | FP |
| **Melanoma not suspected** | FN* | TN |

*In cases where melanoma is not suspected it is likely that a biopsy won’t be taken. However, even in such cases a biopsy may be taken at a later visit and this may confirm melanoma, in which case the case is adjudicated as a FN

** Occasionally, a melanoma may mimic a BCC/SCC or vice versa, and all may be considered in the differential diagnosis. Determining the primary suspicion of the clinician may not be practical. Therefore, cases where any skin cancer is suspected (melanoma/BCC/SCC) will be regarded as ‘melanoma suspected’, and thus if melanoma is found on histopathology these will be categorised as true positive, and if melanoma is not found on histopathology these will be categorised as false positive.

Cases where skin cancer (melanoma/ BCC/SCC) is suspected** AND BCC/SCC is confirmed on pathology will not be considered in this analysis. These are cases where the decision was the correct decision, but does not correspond to the outcome of interest.

**All skin cancers combined analysis**

In this analysis, we are interested in any cases where histopathology confirms any skin cancer (melanoma / other skin cancer) regardless of clinical suspicion AND cases where a biopsy was taken because any skin cancer was suspected (regardless of resulting histopathology)

| **Result of clinical examination** | **Histopathology finding** | | |
| --- | --- | --- | --- |
|  | **Melanoma or**  **other**  **skin cancer** | **Benign condition** |  |
| **Skin cancer suspected (biopsy taken)** | TP | FP |  |
| **Skin cancer not suspected (no biopsy taken)** | FN* | TN |  |

*As for melanoma-specific analysis, even though a biopsy is not taken as a result of this clinical visit, a biopsy may be taken at a later visit which confirms melanoma and the case is adjudicated as a FN. Other skin cancers will not be adjudicated as false negatives.

In cases where the clinical suspicion is not specified, it will be determined by adjudication from pathology request information, patient reports, patient diaries and clinical notes.

Since our primary aim is to quantify unnecessary biopsies for melanoma, the main analysis for the primary objective and all secondary objectives will be melanoma-specific. Supportive analyses for the primary objective and all secondary objectives will also be undertaken based on all skin cancers combined.

1. TABLE SHELLS

Table 1: Basic descriptive statistics of baseline demographic and disease characteristics

| Characteristic | Statistic | Treatment Group | |
| --- | --- | --- | --- |
|  |  | Clinical Surveillance  with MSP | Clinical Surveillance  without MSP |
| Continuous Variable 1 (unit) | n | xx | xx |
|  | Mean (SD) | xxx.x (xx.x) | xxx.x (xx.x) |
|  | Median [IQR] | xxx.x [xxx, xxx] | xxx.x [xxx, xxx] |
| Continuous Variable 2 (unit) | n | xx | xx |
|  | Mean (SD) | xxx.x (xx.x) | xxx.x (xx.x) |
|  | Median [IQR] | xxx.x [xxx, xxx] | xxx.x [xxx, xxx] |
| Categorical Variable 1 | n | xx | xx |
|  | Level 1 | xx (xx.x%) | xx (xx.x%) |
|  | Level 2 | xx (xx.x%) | xx (xx.x%) |
| Categorical Variable 2 | n | xx | xx |
|  | Level 1 | xx (xx.x%) | xx (xx.x%) |
|  | Level 2 | xx (xx.x%) | xx (xx.x%) |
|  | Level 3 | xx (xx.x%) | xx (xx.x%) |

n = count of non-missing values, SD = standard deviation, IQR = inter-quartile range

Table 2: AE’s per type and grade per surgical group

| AE Type | Treatment Group | | | | | | | | | |
| --- | --- | --- | --- | --- | --- | --- | --- | --- | --- | --- |
|  | Clinical Surveillance with MSP | | | | | Clinical Surveillance without MSP | | | | |
|  | Grade 1 | Grade 2 | Grade 3 | Grade 4 | Grade 5 | Grade 1 | Grade 2 | Grade 3 | Grade 4 | Grade 5 |
|  | xx | xx | xx | xx | xx | xx | xx | xx | xx | xx |
|  | xx | xx | xx | xx | xx | xx | xx | xx | xx | xx |
|  | xx | xx | xx | xx | xx | xx | xx | xx | xx | xx |
|  | xx | xx | xx | xx | xx | xx | xx | xx | xx | xx |
|  | xx | xx | xx | xx | xx | xx | xx | xx | xx | xx |
|  | xx | xx | xx | xx | xx | xx | xx | xx | xx | xx |
|  | xx | xx | xx | xx | xx | xx | xx | xx | xx | xx |
|  | xx | xx | xx | xx | xx | xx | xx | xx | xx | xx |
|  | xx | xx | xx | xx | xx | xx | xx | xx | xx | xx |
|  | xx | xx | xx | xx | xx | xx | xx | xx | xx | xx |
| …. |  |  |  |  |  |  |  |  |  |  |

Table 3: False positive (unnecessary biopsy) rate

| Treatment arm | Point Estimate | 95% CI | P-value |
| --- | --- | --- | --- |
| Clinical Surveillance with MSP | xx.x | xx.x, xx.x | - |
| Clinical Surveillance without MSP | xx.x | xx.x, xx.x | - |
| Difference between treatment arm  (MSP – non-MSP) | xx.x | xx.x, xx.x | x.xx |

Table 4: NNE and benign to malignant ratio

| Treatment arm | NNE | | | benign to malignant ratio | | P-value |
| --- | --- | --- | --- | --- | --- | --- |
|  | Point Estimate | 95% CI | Point Estimate | | 95% CI |  |
| Clinical Surveillance with MSP | xx.x | xx.x, xx.x | xx.x | | xx.x, xx.x | - |
| Clinical Surveillance without MSP | xx.x | xx.x, xx.x | xx.x | | xx.x, xx.x | - |
| Difference between treatment arm  (MSP – non-MSP) | xx.x | xx.x, xx.x | xx.x | | xx.x, xx.x | x.xx |

Table 5: Count of patients with all lesions true negative

| Visit Number | Clinical Surveillance  with MSP | Clinical Surveillance  without MSP |
| --- | --- | --- |
| Visit 1 | n | n |
| Visit 2 | n | n |
| : |  |  |
| Visit n | n | n |

Table 6: Count of patients with at least one missed diagnosis

| Visit Number | Clinical Surveillance  with MSP | Clinical Surveillance  without MSP |
| --- | --- | --- |
| Visit 1 | n | n |
| Visit 2 | n | n |
| : |  |  |
| Visit n | n | n |

Table 7: The number of missed diagnoses (false negative) across all patients

| Visit Number | Clinical Surveillance  with MSP | Clinical Surveillance  without MSP |
| --- | --- | --- |
| Visit 1 | n | n |
| Visit 2 | n | n |
| : |  |  |
| Visit n | n | n |

Table 8: The total number of true positive findings across all patients

| Visit Number | Clinical Surveillance  with MSP | Clinical Surveillance  without MSP |
| --- | --- | --- |
| Visit 1 | n | n |
| Visit 2 | n | n |
| : |  |  |
| Visit n | n | n |
