# Supplementary material for: Study protocol for a randomised controlled trial to evaluate the use of melanoma surveillance photography to the Improve early detection of MelanomA in ultra-hiGh and high-risk patiEnts (the IMAGE trial)
Source: Trials. 2023 Mar 29;24:236. doi: 10.1186/s13063-023-07203-5 (PMC10061902; doi:10.1186/s13063-023-07203-5)
Supplement: Supplementary file 3 — Additional file 3. Supplementary Table. [file 13063_2023_7203_MOESM3_ESM.docx]

## Supplementary Table

| Data category | Information |
| --- | --- |
| Primary registry and trial identifying number | ClinicalTrials.gov NCT04385732 |
| Date of registration in primary registry | 04 Jan 2020 |
| Secondary identifying numbers | 02.19 IMAGE |
| Source(s) of monetary or material support | the Medical Research Future Fund – Targeted Health System and Community Organisation Research competitive grant (RGMS ID 1175082 |
| Primary sponsor | Monash University |
| Secondary sponsor(s) | Australian Government |
| Contact for public queries | [image@masc.org.au](mailto:image@masc.org.au) |
| Contact for scientific queries | A/Prof Victoria Mar, [victoria.mar@monash.edu](mailto:victoria.mar@monash.edu) |
| Public title | Melanoma Surveillance Photography (MSP) to Improve Early Detection of  Melanoma in Ultra-high and High Risk Patients |
| Scientific title | Melanoma Surveillance Photography (MSP) to Improve Early Detection of  Melanoma in Ultra-high and High Risk Patients |
| Countries of recruitment | Australia |
| Health condition(s) or problem(s) studied | Melanoma |
| Intervention(s) | Active comparator: Melanoma surveillance photography + clinical surveillance according to guidelines |
|  | Control comparator: Clinical surveillance according to guidelines |
| Key inclusion and exclusion criteria | Ages eligible for study: ≥18 years Sexes eligible for study: both Accepts healthy volunteers: no |
|  | Inclusion criteria: Australian adult patients within 24 months of primary melanoma diagnosis at high or very high risk of subsequent primary melanoma and with multiple naevi |
|  | Exclusion criteria: Previously under active surveillance with total body photography; stage IV metastatic melanoma; ocular melanoma, mucosal melanoma; participation in another clinical trial or study involving MSP |
| Study type | Interventional |
|  | Allocation: randomised intervention model. No blinding. |
|  | Primary purpose: prevention |
|  | Study phase: N/A |
| Date of first enrolment | March 2020 |
| Target sample size | 580 |
| Recruitment status | Recruiting |
| Primary outcome(s) | Diagnostic performance of surveillance for melanoma |
| Key secondary outcomes | Additional diagnostic performance outcomes for melanoma; diagnostic performance outcomes for keratinocyte lesions; quality of life; patient anxiety and acceptability evaluation; cost-effectiveness of MSP; healthcare budget impact of MSP |
